# Quantification of O-(2-[^18^F]fluoroethyl)-L-tyrosine kinetics in glioma

**DOI:** 10.1186/s13550-018-0418-0

**Published:** 2018-07-31

**Authors:** Thomas Koopman, Niels Verburg, Robert C. Schuit, Petra J. W. Pouwels, Pieter Wesseling, Albert D. Windhorst, Otto S. Hoekstra, Philip C. de Witt Hamer, Adriaan A. Lammertsma, Ronald Boellaard, Maqsood Yaqub

**Affiliations:** 1 Department of Radiology and Nuclear Medicine, VU University Medical Center, PO Box 7057, 1007 MB Amsterdam, The Netherlands; 2 Neurosurgical Center Amsterdam, VU University Medical Center, Amsterdam, The Netherlands; 3 Brain Tumor Center Amsterdam, Amsterdam, The Netherlands; 4 Department of Pathology, VU University Medical Center, Amsterdam, The Netherlands; 5 Department of Pathology, Princess Máxima Center for Pediatric Oncology, Utrecht, The Netherlands; 6 Department of Pathology, University Medical Center Utrecht, Utrecht, The Netherlands; 7 Department of Nuclear Medicine and Molecular Imaging, University Medical Center Groningen, Groningen, The Netherlands

**Keywords:** FET, Quantification, Kinetic modelling, SUV, SUVR

## Abstract

**Background:**

This study identified the optimal tracer kinetic model for quantification of dynamic O-(2-[^18^F]fluoroethyl)-L-tyrosine ([^18^F]FET) positron emission tomography (PET) studies in seven patients with diffuse glioma (four glioblastoma, three lower grade glioma). The performance of more simplified approaches was evaluated by comparison with the optimal compartment model. Additionally, the relationship with cerebral blood flow—determined by [^15^O]H_2_O PET—was investigated.

**Results:**

The optimal tracer kinetic model was the reversible two-tissue compartment model. Agreement analysis of binding potential estimates derived from reference tissue input models with the distribution volume ratio (DVR)-1 derived from the plasma input model showed no significant average difference and limits of agreement of − 0.39 and 0.37. Given the range of DVR-1 (− 0.25 to 1.5), these limits are wide. For the simplified methods, the 60–90 min tumour-to-blood ratio to parent plasma concentration yielded the highest correlation with volume of distribution *V*_*T*_ as calculated by the plasma input model (*r* = 0.97). The 60–90 min standardized uptake value (SUV) showed better correlation with *V*_*T*_ (*r* = 0.77) than SUV based on earlier intervals. The 60–90 min SUV ratio to contralateral healthy brain tissue showed moderate agreement with DVR with no significant average difference and limits of agreement of − 0.24 and 0.30. A significant but low correlation was found between *V*_*T*_ and CBF in the tumour regions (*r* = 0.61, *p* = 0.007).

**Conclusion:**

Uptake of [^18^F]FET was best modelled by a reversible two-tissue compartment model. Reference tissue input models yielded estimates of binding potential which did not correspond well with plasma input-derived DVR-1. In comparison, SUV ratio to contralateral healthy brain tissue showed slightly better performance, if measured at the 60–90 min interval. SUV showed only moderate correlation with *V*_*T*_. *V*_*T*_ shows correlation with CBF in tumour.

**Electronic supplementary material:**

The online version of this article (10.1186/s13550-018-0418-0) contains supplementary material, which is available to authorized users.

## Background

Since its introduction in 1999 [1], the amino acid tracer O-(2-[^18^F]fluoroethyl)-L-tyrosine ([^18^F]FET) is increasingly used to image glioma [2]. Because [^18^F]FET is not incorporated into proteins, it is a tracer for amino acid transport rather than for protein synthesis rate [1, 3]. [^18^F]FET positron emission tomography (PET) has shown its added value to magnetic resonance imaging (MRI) for several clinical problems regarding brain tumours, such as prognosis assessment, delineation of tumour extent and glioma grading [4].

The most extensive quantitative analysis of a PET tracer is based on dynamic PET scans in combination with plasma input-based pharmacokinetic modelling [5]. For large clinical studies, such an extensive analysis is not feasible; tracer uptake needs to be quantified using simplified measures. For example, the standardized uptake value (SUV) interval of 20–40 min post-injection is currently recommended for clinical reading in European Association of Nuclear Medicine and German guidelines [6, 7]. Simplified approaches are not only affected by regulation of specific amino acid transporters—the primary parameter of interest—but also by the blood flow and plasma concentration, which is in turn affected by the biodistribution, tracer metabolism and uptake in blood cells. It is of interest to quantify these effects to gain a better understanding of the accuracy of a simplified measure and its reliability.

In the current literature, we identified five studies which used pharmacokinetic modelling to quantify uptake of the tracer in the brain: two preclinical studies [8, 9] and three human studies [10–12]. The human studies all used an image-derived input function. Furthermore, we found only one study where metabolite concentration in plasma was measured [13]. The tracer kinetics of [^18^F]FET in glioma patients are expected to be in line with preclinical research, but validation of kinetic models is needed. The aim of this study was therefore to identify the optimal metabolite-corrected plasma input model for the quantification of [^18^F]FET kinetics. In addition, reference tissue input models and several simplified methods were validated in terms of their agreement with full kinetic analysis results. Lastly, the relationship of the methods and parameters with blood flow was investigated using [^15^O]H_2_O PET data.

## Methods

### Subjects and study protocol

The study population consisted of seven patients with diffuse glioma from an ongoing patient study [14]. Each patient gave written informed consent prior to inclusion. This study has been performed in accordance with the Declaration of Helsinki, approved by the Medical Ethics Committee of the VU University Medical Center and registered in the Netherlands National Trial Register (www.trialregister.nl, unique identifier NTR5354, registration date 4th of August 2015). The age of the patients ranged from 22 to 69 years. All gliomas were newly diagnosed and selected for resective surgery. Imaging was preoperatively performed. Based on histology of biopsies taken before surgery—but after imaging—each glioma was classified according to World Health Organization (WHO) criteria as lower grade (WHO II-III) or glioblastoma (WHO IV) [15]. Four patients presented with glioblastoma, three with lower grade glioma. See Additional file 1: Table S1 for more details.

The patients were required to fast at least 4 h before undergoing the imaging protocol. T1-weighted gadolinium-enhanced (T1G) and FLAIR sequences were acquired on an Achieva whole-body 3.0T MR-scanner (Philips Healthcare, Best, the Netherlands). Details of the MR sequences are described in the Additional file 1. Two dynamic PET scans were acquired on either a Gemini TF-64 PET/CT or an Ingenuity TF PET/CT (Philips Healthcare, Best, the Netherlands). Each scan started with a low-dose computed tomography (CT) scan (30 mAs, 120 kVp) for attenuation and scatter correction purposes. A bolus of 800 MBq [^15^O]H_2_O was administered at the start of the first scan with a venous line, and emission scans were acquired in list mode for 10 min. An arterial line in the opposite arm was used for continuous sampling using an on-line blood sampler (Comecer Netherlands, Joure, the Netherlands). Manual arterial samples were collected at 5, 7 and 9 min. A 90-min dynamic scan was then acquired on the same system after a bolus of 200 MBq [^18^F]FET. [^18^F]FET was produced following the method earlier described [16]. The radiochemical purity was > 98% and the specific radioactivity > 18.5 GBq μmol^−1^. Arterial blood was continuously sampled, and manual samples were taken at 5, 10, 20, 40, 60, 75 and 90 min. The line-of-response row-action maximum likelihood algorithm (LOR-RAMLA) as provided by the scanner manufacturer was used for reconstruction of the scans into 26 time frames (1 × 10, 8 × 5, 4 × 10, 2 × 15, 3 × 20, 2 × 30, 6 × 60 s) and 22 time frames (1 × 15, 3 × 5, 3 × 10, 4 × 60, 2 × 150, 2 × 300, 7 × 600 s), respectively, both with an isotropic voxel size of 2 mm.

The measured arterial whole blood curve was calibrated using manual arterial samples. Then, metabolite-corrected plasma curves were constructed from the whole blood curve by correcting for the plasma to whole blood ratio and labelled metabolites concentration. The parent fractions were fitted with a Hill function [17]. Concentration of both polar and non-polar metabolites was determined using solid phase extraction in combination with high-performance liquid chromatography. More details on the blood measurements can be found in the Additional file 1.

### Image processing and segmentation

The reconstructed PET images were checked frame by frame for movement and corrected accordingly. Affected time frames were rigidly coregistered to the attenuation scan using the generic multi-modality registration setup from Vinci (version 2.56.0, Max Planck Institute for Metabolism Research). However, if patient movement was more than 5 mm, the affected time frames were reconstructed after re-aligning the attenuation scan. The newly reconstructed frames were coregistered to the original attenuation scan.

Tumour volumes were delineated on the MR images by a resident in neurosurgery with ample experience in imaging characteristics of patients with glial tumours. MR sequences were selected based on grade. Lower grade glioma was delineated using the FLAIR sequence; glioblastoma was delineated on T1G. These delineations were transferred to the dynamic PET scan after rigid coregistration—using the same registration setup—of the MR scan to the CT scan. Volume of the tumour delineations ranged from 25.2 to 100.8 cm^3^. In order to minimise heterogeneity, the MR-based delineations were divided into three volumes of interest (VOI) based on the 33rd and 67th percentiles of the 20–40 min [^18^F]FET uptake value. These VOIs were labelled low, medium or high uptake. For the reference region, a spherical VOI with 14 mm radius was placed at the mirror location of the tumour on the contralateral side, encompassing white and grey matter tissue. In addition, two more spherical VOIs of the same volume were placed at the contralateral side, not overlapping the reference region. Together with the reference region, these form the VOIs of presumed non-tumour (healthy) brain tissue and were used to investigate the pharmacokinetics in healthy tissue.

### Kinetic analysis of [^15^O]H_2_O

Parametric maps of cerebral blood flow (CBF) were constructed from the [^15^O]H_2_O PET scans and the plasma input functions using the basis function implementation of the standard single-tissue compartment model [18]. The CBF maps were coregistered to the summed [^18^F]FET image, and the average value within each VOI was calculated. CBF was normalised to the same reference region to calculate the CBF-ratio.

### Kinetic analysis of [^18^F]FET

Time-activity curves (TACs) were generated by projecting the VOIs on the dynamic [^18^F]FET PET images. These TACs were analysed with several pharmacokinetic plasma input models: the reversible single-tissue compartment model (1T2k_Vb_), the irreversible two-tissue compartment model (2T3k_Vb_) and the reversible two-tissue compartment model (2T4k_Vb_) [19]. All models included an additional fit parameter for fractional blood volume (Vb) and therefore included both the whole blood and the metabolite-corrected plasma curve as input functions. The input functions were corrected for delay using a whole brain TAC. All models were fitted using weighted non-linear regression [20]. Parameter errors were calculated as standard deviation, to estimate the reliability of the fitted kinetic parameter. To identify the optimal model, the fits of the pharmacokinetic plasma input models were evaluated visually and with the Akaike information criterion [21]. Main kinetic parameters of interest were the volume of distribution (*V*_*T*_) for the reversible models, the influx rate constant (*K*_*i*_) for the irreversible model and the rate constant from plasma to tissue (*K*_1_). The relationship of these parameters with CBF was investigated using Pearson’s correlation coefficient (*r*). A *p* value less than 0.05 was considered significant. *K*_1_ was also divided by CBF to calculate the extraction fraction. The distribution volume ratio (DVR) was calculated by normalising the *V*_*T*_ using the *V*_*T*_ of reference region. The nondisplaceable binding potential, BP_ND_ [22], was then derived by BP_ND_ = DVR-1 and used to validate BP_ND_ obtained using reference tissue input models (next paragraph).

Performance of both the full reference tissue model (FRTM) [23, 24] and the simplified reference tissue model (SRTM) [25] was investigated. The advantage of reference tissue input models is that no arterial input function is needed. Instead, a reference region is used as indirect input function, in this case, the contralateral reference region. In this study, we assessed agreement between FRTM or SRTM-derived BP_ND_ vs plasma input model-derived DVR-1 and, similarly, *R*_1_ vs plasma input model-derived K_1_-ratio (K_1_ normalised to reference region) using Bland-Altman [26] analysis. The relationship of BP_ND_ and *R*_1_ with the CBF-ratio was also investigated.

We calculated SUV for intervals 20–40 min (SUV^20–40^), 40–60 min (SUV^40–60^) and 60–90 min (SUV^60–90^) and calculated correlation with *V*_*T*_. We also calculated tumour-to-blood ratios (TBlR) to investigate whether this would be a possible surrogate of *V*_*T*_. Two variants were considered: ratio to whole blood activity (TBlR_WB_) and ratio to parent plasma activity (TBlR_PP_). Furthermore, relationship with CBF for all the above parameters was investigated. The SUV ratio (SUVR, SUV normalised to reference region; also known as tumour-to-brain or tumour-to-normal ratio) was also calculated for these three intervals. Agreement with DVR was evaluated using Bland-Altman analysis, and correlation with CBF-ratio was determined.

## Results

**Fig. 1:**
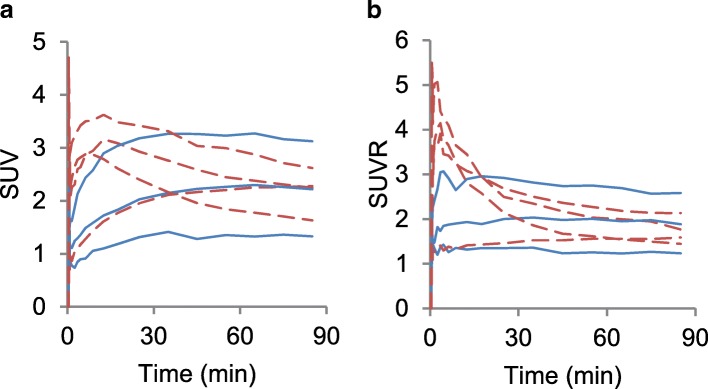
SUV (**a**) and SUVR (**b**) curves of the high [^18^F]FET uptake VOI of each patient. Solid lines are lower grade gliomas, and dashed lines are glioblastoma

One of the lower grade glioma patients, patient two, showed very little uptake in the tumour yet could be visually distinguished based on the SUV^20–40^, see Additional file 1: Figure S1. Figure 1 illustrates this and shows the SUV and SUVR over time for the high uptake VOIs. All except one tumour, from patient three, show the typical curve pattern generally associated with their grade [2]. During acquisition of the [^15^O]H_2_O PET scan of patient six, there were problems with the measurement of the arterial blood activity. CBF could therefore not be quantified for this patient. Two patients had moved during the dynamic [^18^F]FET PET scan, one had moved approximately 3 mm and the other 15 mm, both after at least 20 min. Both scans were corrected as described above.

**Fig. 2:**
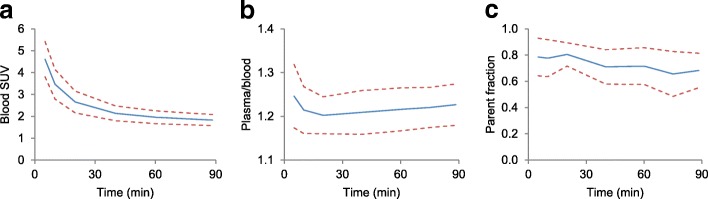
Data from manual blood samples, showing the whole blood activity concentration over time corrected for injected dose and patient weight (**a**), the ratio of activity concentration in plasma over activity concentration in whole blood (**b**) and the percentage parent compound in the samples (**c**). Solid lines are the average, and dashed lines show the average ± SD over all patients

Figure 2 shows results from the manual blood sample measurements for the [^18^F]FET scans. The plasma to whole blood ratio is stable at an average of 1.22 ± 0.05 (standard deviation between patients). The parent fraction of [^18^F]FET was 79 ± 14% at time of the first manual blood sample (5 min post-injection) and decreased slowly to 68 ± 13% at 90 min post-injection.

**Fig. 3:**
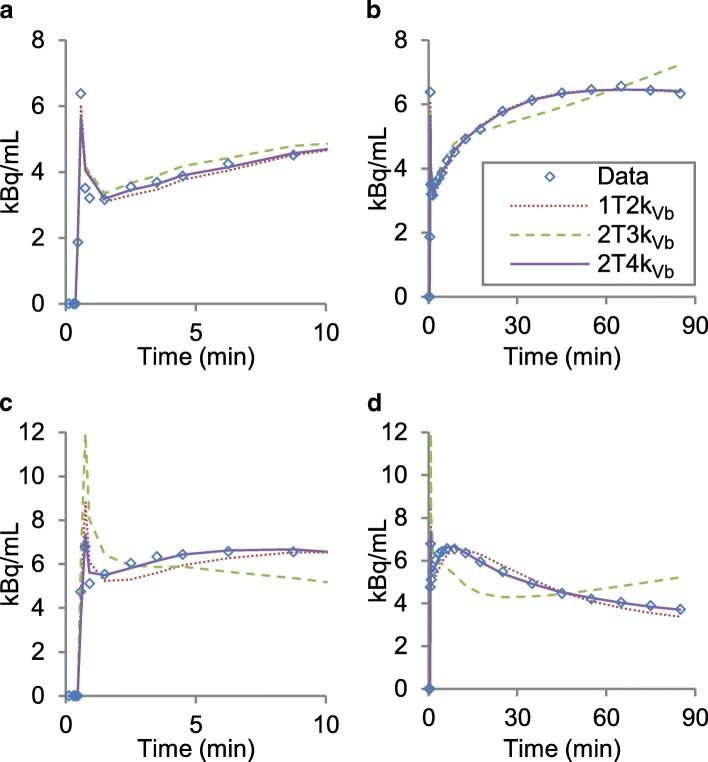
Typical example of a TAC with fits of the three models: 1T2k_Vb_ dotted line, 2T3k_Vb_ dashed line and 2T4k_Vb_ solid line. The TAC of the high uptake VOI of patient 5, lower grade glioma; the first 10 min of the TAC (**a**) and the whole 90 min (**b**). The TAC of the high uptake VOI of patient 6, glioblastoma; the first 10 min of the TAC (**c**) and the whole 90 min (**d**)

Visual assessment of the fits showed that the irreversible model was not able to fit the tumour TACs. Figure 3 shows a typical example. The Akaike information criterion confirmed this finding and showed a preference for the 2T4k_Vb_ model in 95% (20/21) of the fitted TACs; for the other 5% (1/21), the 1T2k_Vb_ model was preferred. As such, the model preference seems independent of both uptake and grade as determined by histological assessment. In contralateral (healthy) brain tissue, the 2T4k_Vb_ model was preferred in 52% (11/21) of the regions and the 1T2k_Vb_ model in the other 48% (10/21). Correlation for *V*_*T*_ in the tumour regions as derived from 2T4k_Vb_ and 1T2k_Vb_ was very high (*r* = 0.99); however, agreement analysis showed a significant difference for estimated *V*_*T*_ of 0.08 (9%), as shown in the Bland Altman plot in Additional file 1: Figure S2. The two-tissue reversible model was therefore used as reference for further analyses.

A significant but low correlation was found between *V*_*T*_ and CBF in the tumour regions (*r* = 0.61, *p* = 0.007); a scatter plot is shown in Additional file 1: Figure S3. There was no correlation between *K*_1_ values of [^18^F]FET and CBF in the tumour regions (*r* = − 0.018, *p* = 0.93), Additional file 1: Figure S4. The calculated extraction fractions showed little variation in the non-tumour regions with a mean value of 0.071 and a standard deviation of 0.024. Extraction fraction in the tumour regions was higher with a mean value of 0.17 and a standard deviation of 0.13. A scatter plot of extraction fraction against CBF in both tumour and healthy regions is shown in Additional file 1: Figure S5.

**Fig. 4:**
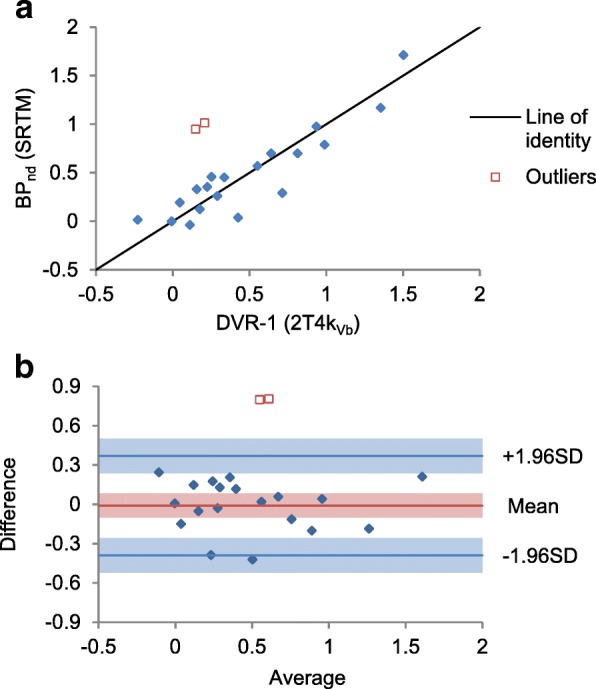
Agreement between BP_ND_ from SRTM and the DVR-1 from the 2T4k_Vb_ model. Scatter plot (**a**) and Bland Altman plot (**b**). Shaded areas are 95% confidence intervals

Agreement between the estimated BP_ND_ from SRTM and DVR-1 from the 2T4k_Vb_ is shown in Fig. 4. Two outliers were identified, the low and medium uptake VOIs of patient two. The error of these BP_ND_ estimates was very high (standard deviations of 10.6 and 31.6). If we disregard these outliers, the limits of agreement are − 0.39 and 0.37 (range DVR-1 − 0.25 to 1.5). Agreement of *R*_1_ with *K*_1_-ratio from 2T4k_Vb_ was poor with an average difference of − 0.90 and limits of agreement of − 3.23 and 1.44 (range *K*_1_-ratio 0.85 to 4.8). BP_ND_ showed significant correlation with the CBF-ratio (*r* = 0.83, *p* < 0.001), and *R*_1_ showed a significant but low correlation with the CBF-ratio (*r* = 0.52, *p* = 0.039); the scatterplots are shown in Additional file 1: Figure S6. FRTM estimates of BP_ND_ mostly agreed with SRTM; however, several additional outliers were seen with high parameter error of BP_ND_.

**Fig. 5:**
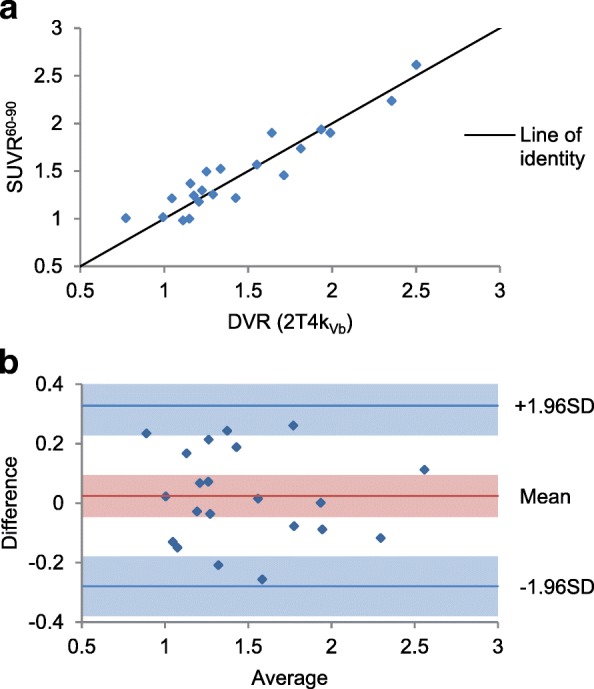
Agreement between SUVR^60–90^ and the DVR from the 2T4k_Vb_ model. Scatter plot (**a**) and Bland Altman plot (**b**). Shaded areas are 95% confidence intervals

Correlation between SUV^20–40^ and *V*_*T*_ was significant but low (*r* = 0.62, *p* < 0.001); the scatter plot is shown in Additional file 1: Figure S7. Correlation with *V*_*T*_ was higher for later time intervals, and this was also seen for TBlR_WB_ and TBlR_PP_ and for the correlations between SUVR and DVR. Correlation with *K*_1_ was higher for earlier time intervals. Correlation coefficients are given in Table 1. The agreement between SUVR and DVR showed a similar pattern, where the SUVR for later time intervals show better agreement with DVR as calculated with the 2T4k_Vb_ model. SUVR^60–90^ showed limits of agreement of − 0.27 and 0.34, see Fig. 5, while limits of agreement for SUVR^20–40^ were − 0.52 and 0.85 (range DVR 0.75 to 2.5).

**Table 1:**
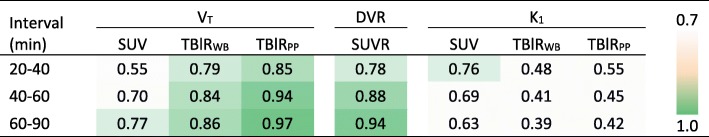
Pearson correlation *r* between SUV-based measures and kinetic parameters from 2T4k_Vb_

Neither SUV nor TBlR_WB_ showed significant correlation with CBF. In contrast, TBlR_PP_ did show significant correlation with CBF and the correlation increased at later time intervals. For the 60–90 min interval, the correlation coefficient was *r* = 0.63, *p* = 0.005. TBlR_PP_ also showed agreement with *V*_*T*_ with limits of agreement of − 0.17 and 0.19 (range *V*_*T*_ 0.53 to 2.1) and without significant bias. SUVR showed significant correlation with the CBF-ratio; for all time intervals, the correlation was higher than 0.85. It was highest for the 20–40 min interval at 0.91, *p* < 0.001.

## Discussion

The aim of this study was to derive the optimal plasma input kinetic model for dynamic [^18^F]FET PET studies and to validate performance of simplified methods. Therefore, various metabolite-corrected plasma input models were evaluated, and the optimal model was determined. Next, the optimal model was used to assess the agreement of various simplified methods with the optimal model including approaches often used in [^18^F]FET PET studies in glioma.

The optimal plasma input kinetic model was found to be the reversible two-tissue compartment model with fitted blood volume fraction. The model preference based on the Akaike criterion was clear for the tumour regions, where only 5% could be better fitted with the single-tissue compartment model. These data indicate that the model preference is independent of tumour grade or curve pattern, although there are too few data to substantiate this in this study. Healthy tissue regions were best fitted by the reversible two-tissue compartment model in half of the cases and by a single-tissue compartment model in the other half. Use of the single-tissue compartment model resulted in systematically lower estimates of *V*_*T*_: in tumour regions with an average difference of − 9% and in healthy regions with an average difference of − 7%. Based on the fits of all target and reference tissue TACs, we concluded that the two-tissue compartment model is most suitable for the further evaluations.

Fully quantitative pharmacokinetic models require arterial plasma input functions. In this study, manual arterial samples were used to correct for the labelled metabolite concentration. In an earlier report, results of metabolite measurements showed low fractions (5% at 5 min post-injection, 13% at 120 min post-injection), suggesting rapid excretion of labelled metabolites by the kidneys [13]. In our study, the results from the manual arterial blood samples showed a larger fraction of metabolites in blood (21% at 5 min post-injection, 32% at 90 min post-injection). In an effort to investigate the effect of correction for the labelled metabolites, we fitted a 2T4k_Vb_ model with a whole plasma input function. Estimates of *V*_*T*_ were on average 39% lower. Yet, estimates of DVR were the same on average. Therefore, the impact of using metabolite-corrected input functions versus whole plasma input function on the validation of reference region-based models or simplified methods is minimal.

The results on the relationship with blood flow showed a significant correlation of *V*_*T*_ with CBF, but the correlation was low. As *V*_*T*_ represents a perfusion independent estimate of tracer uptake, the observed correlation is likely due to physiological coincidence of both increased amino acid utilisation and perfusion. This makes it impossible to draw conclusions about perfusion dependence of the simplified methods. The absence of correlation between *K*_1_ and CBF suggests that the extraction fraction is highly variable between patients. Indeed, the variation in the calculated extraction fractions is relatively high in the tumour regions across the patients. This could be the consequence of different levels of transporter expression or may be due to differences in blood-brain barrier breakdown.

Agreement analysis on the simplified reference tissue model BP_ND_ vs plasma input-derived DVR-1 showed wide limits of agreement. As such, BP_ND_ seems a poor surrogate for this parameter. Agreement for *R*_1_ vs the *K*_1_-ratio was poor as well. The full reference tissue model showed no different results from the simplified reference tissue model, except for a few additional outliers. The poor performance of the reference tissue input model might be due to violated assumptions, making the model invalid. One of the assumptions is that both reference and target regions can be represented by a single-tissue compartment model. For half of these data, both regions are better described by a two-tissue compartment model; for the other half, the target region is better described by two tissue compartments while the normal regions are best described by a single-tissue compartment. The expected error from the first violation is minor, while the second violation can lead to a 10% bias [27]. Another possible source of error is non-negligible blood volume contribution. Moreover, use of reference tissue input models requires that the transport across the blood-brain barrier, represented by *K*_1_/*k*_2_ ratio, is equivalent between target (tumour) and reference regions. In case of gliomas, tracer uptake in the tumour can be affected by disruptions of the blood-brain barrier. Consequently, use of reference tissue input models may not be valid for dynamic [^18^F]FET brain studies.

The TBlR_PP_^60–90^ showed good agreement with *V*_*T*_. A disadvantage of the TBlR_WB_ and TBlR_PP_ is the requirement of blood samples and, for TBlR_PP_, the need for metabolite measurements. However, their correlation results suggest that plasma clearance effects (and thus variability in input functions between subjects) seem the largest contributor to SUV variability. If we convert the correlation results to coefficients of determination, we see that 94% of the variability in TBlR_PP_^60–90^ can be explained by the variability in V_T_. This is encouraging for the use of SUVR, which largely corrects for variability of the input functions between patients.

For SUV, TBlR_WB_ and TBlR_PP_ uptake intervals later than the currently recommended 20–40 min show better correlation with *V*_*T*_. Correlation was lowest for SUV^20–40^ and highest for TBlR_PP_^60–90^. Furthermore, from the time activity curves, it becomes clear that the uptake value of the tumours is still changing during the 20–40 min interval, see Fig. 1. A possible downside of early static imaging might be that variability in uptake time will lead to variability in SUV. In contrast, the SUVR curves of four patients are relatively stable during this period. Three patients, however, show a variable SUVR at the 20–40 min interval, which becomes more constant at later times. The agreement of SUVR with DVR also improves at later time intervals. The size of this improvement is clearly illustrated by the limits of agreement, which are more than twice as wide for the 20–40 min interval. In terms of limits of agreement, SUVR^60–90^ showed a slightly better agreement with DVR than SRTM. Just like for SRTM, a possible source of error is the blood-volume fraction, especially in case of blood-brain barrier disruption. To conclude, early time point imaging (20–40 min post-injection) is usually applied and preferred in a clinical setting. A downside to static imaging is that the time activity curve pattern cannot be assessed, which has been shown to be helpful in determining the grade of glioma. Furthermore, when non-invasive quantification is required, it is recommended to use SUVR at later time points (60–90 min post-injection). When studies are designed to measure changes (longitudinally or after intervention), use of TBlR_WB_ and TBlR_PP_ would be recommended, because of the better agreement with plasma input-derived *V*_*T*_ and the ability of compensating for inter-subject variability of the input function. Further studies are needed to investigate whether this improved quantification also improves the clinical value.

It must be noted that the small sample size of this study requires appropriate caution in the interpretation of the results presented here. The complexity of compartmental modelling with metabolite corrected plasma input function do not enable large study cohorts, yet compartmental modelling is an important step in the evaluation of tracer kinetics and its implications for more simplified approaches. The results of this study only apply to regional analyses, i.e. based on the mean signal of a VOI. Thus, relationships between parameters within a scan cannot be adequately investigated, because the number of data points (VOIs) per scan was limited. Voxel-based methods enable such analysis but require further evaluation due to higher noise levels in voxel-based signals.

## Conclusion

In this study, we derived that the two-tissue reversible plasma input model with fitted blood volume fraction is the optimal plasma input model to describe the kinetics of [^18^F]FET in glioma patients. Furthermore, use of reference tissue input models and simplified methods, such as SUV and SUVR, was validated. BP_ND_ results obtained with reference tissue input models did not correspond well with plasma input-derived DVRs, possibly due to violation of the reference tissue model assumptions. SUVR showed slightly better agreement with DVR than SRTM-derived BP_ND_. SUV only moderately correlated with *V*_*T*_ with the best correspondence at later uptake time intervals (60–90 min post-injection). The results of the study suggest that later time point imaging (60–90 min post-injection) outperforms currently recommended uptake time (20–40 min post-injection) in terms of quantitative value, i.e. correlation with *V*_*T*_ and DVR.

## Additional files

Additional file 1: Details MR sequences. Details blood sample measurements. **Table S1.** Patient details. **Figure S1.** Transaxial views of the tumours on 20–40 min standardised uptake value maps of [^18^F]FET. **Figure S2.** Scatter (A) and Bland-Altman plot (B) of volume of distribution, *V*_*T*_, calculated with the 1T2k_Vb_ model versus the 2T4k_Vb_ model. Shaded areas are 95% confidence intervals. **Figure S3.** Scatterplot of volume of distribution (*V*_*T*_) versus cerebral blood flow (CBF) (A). The same plot with each patient indicated separately, connecting low, medium and high VOIs with lines (B). CBF data was not available for patient 6. **Figure S4.** Scatterplot of *K*_1_ versus cerebral blood flow (CBF) (A). The same plot with each patient indicated separately, connecting low, medium and high VOIs with lines (B). CBF data was not available for patient 6. **Figure S5.** Scatterplot of extraction versus cerebral blood flow (CBF). **Figure S6.** Scatterplots of simplified reference tissue model estimates of binding potential (BP_ND_) (A) and K_1_-ratio (*R*_*1*_) (B) against the cerebral blood flow ratio (CBF-ratio). **Figure S7.** Scatter plot of SUV^20–40^ versus the volume of distribution (*V*_*T*_) calculated with the 2T4k_Vb_ model. (PDF 237 kb)

Additional file 2: Patient information, TACs, AIFs. (XLSX 443 kb)

## Abbreviations

[^18^F]FET: O-(2-[^18^F]fluoroethyl)-L-tyrosine
1T2k_Vb_: Reversible single-tissue compartment model with blood volume fraction
2T3k_Vb_: Irreversible two-tissue compartment model with blood volume fraction
2T4k_Vb_: Reversible two-tissue compartment model with blood volume fraction
BP_ND_: Nondisplaceable binding potential
CBF: Cerebral blood flow
CT: Computed tomography
DVR: Distribution volume ratio
FRTM: Full reference tissue model
*K*_1_: Rate constant from blood to tissue
*K*_*i*_: Influx rate constant for an irreversible model
MRI: Magnetic resonance imaging
PET: Positron emission tomography
SRTM: Simplified reference tissue model
SUV: Standardized uptake value
SUVR: Standardized uptake value-ratio to reference region
TBlR_PP_: Tumour-to-blood ratio; ratio to parent plasma activity concentration
TBlR_WB_: Tumour-to-blood ratio; ratio to whole blood activity concentration
VOI: Volume of interest
*V*_*T*_: Volume of distribution
WHO: World Health Organization
