# Supplementary material for: Quantification of O-(2-[18F]fluoroethyl)-L-tyrosine kinetics in glioma
Source: EJNMMI Res. 2018 Jul 31;8:72. doi: 10.1186/s13550-018-0418-0 (PMC6068050; doi:10.1186/s13550-018-0418-0)
Supplement: Supplementary file 1 — Details MR sequences. Details blood sample measurements. Table S1. Patient details. Figure S1. Transaxial views of the tumours on 20–40 min standardised uptake value maps of [18F]FET. Figure S2. Scatter (A) and Bland-Altman plot (B) of volume of distribution, VT, calculated with the 1T2kVb model versus the 2T4kVb model. Shaded areas are 95% confidence intervals. Figure S3. Scatterplot of volume of distribution (VT) versus cerebral blood flow (CBF) (A). The same plot with each patient indicated separately, connecting low, medium and high VOIs with lines (B). CBF data was not available for patient 6. Figure S4. Scatterplot of K1 versus cerebral blood flow (CBF) (A). The same plot with each patient indicated separately, connecting low, medium and high VOIs with lines (B). CBF data was not available for patient 6. Figure S5. Scatterplot of extraction versus cerebral blood flow (CBF). Figure S6. Scatterplots of simplified reference tissue model estimates of binding potential (BPND) (A) and K1-ratio (R1) (B) against the cerebral blood flow ratio (CBF-ratio). Figure S7. Scatter plot of SUV20–40 versus the volume of distribution (VT) calculated with the 2T4kVb model. (PDF 237 kb) [file 13550_2018_418_MOESM1_ESM.pdf]

Details MR sequences

The MR sequences were acquired on an Achieva whole-body 3.0T MR-scanner (Philips), equipped with the standard head coil. Each patient was scanned with a sagittal 3D FLAIR sequence (TR/TE/TI (inversion time) 4800/279/1650ms, acquired voxel size 1.12x1.12x1.12 mm, reconstructed voxel size 1.04x1.04x0.56 mm), and a sagittal 3D T1-weighted gadolinium-enhanced (T1G) sequence (TR/TE/TI/flip angle 7/3/950ms/12°, acquired voxel size 0.98x0.98x1.0 mm, reconstructed voxel size 0.89x0.89x1.0 mm).

Details blood sample measurements

1 mL of plasma was diluted with 2 mL of water and loaded onto an activated Sep-Pak tC18. The cartridge was first washed with 3 mL of water and then eluted with 1 mL of methanol followed by 2 mL of water. Radioactivity in all three fractions (plasma, water, methanol/water) was quantified. Radioactivity in the plasma and water fraction represented the polar radiolabelled metabolites of [^{18}F]FET. Radioactivity in the methanol/water fraction was further analysed with HPLC with radioactivity detection. The eluate was mixed and injected onto a gradient high performance liquid chromatography system. Stationary phase Phenomenex LUNA C18, 5µm. 250*10 mm. Mobile phase: flow 3 ml/min. A = acetonitrile, B = 0.01 M phosphoric acid. Gradient: t=0, 90%B; t=10, 40%B; t=12, 40%B, t=12.5, 90%B; t=15, 90%B. The HPLC eluate was monitored in series for ultraviolet and radioactivity. Fractions were collected and counted for radioactivity using a gamma counter. A radiochromatogram was reconstructed in Microsoft Excel.

Supplemental Figures and tables

TABLE S1

Patient details

Patient	Glioma	Grade	IDH1 status	1p19q codeletion
1	Glioblastoma	IV	wild type	-
2	Astrocytoma	II	mutant	no
3	Glioblastoma	IV	mutant	-
4	Oligodendrocytoma	II	mutant	yes
5	Astrocytoma	II	mutant	no
6	Glioblastoma	IV	wild type	-
7	Glioblastoma	IV	wild type	-

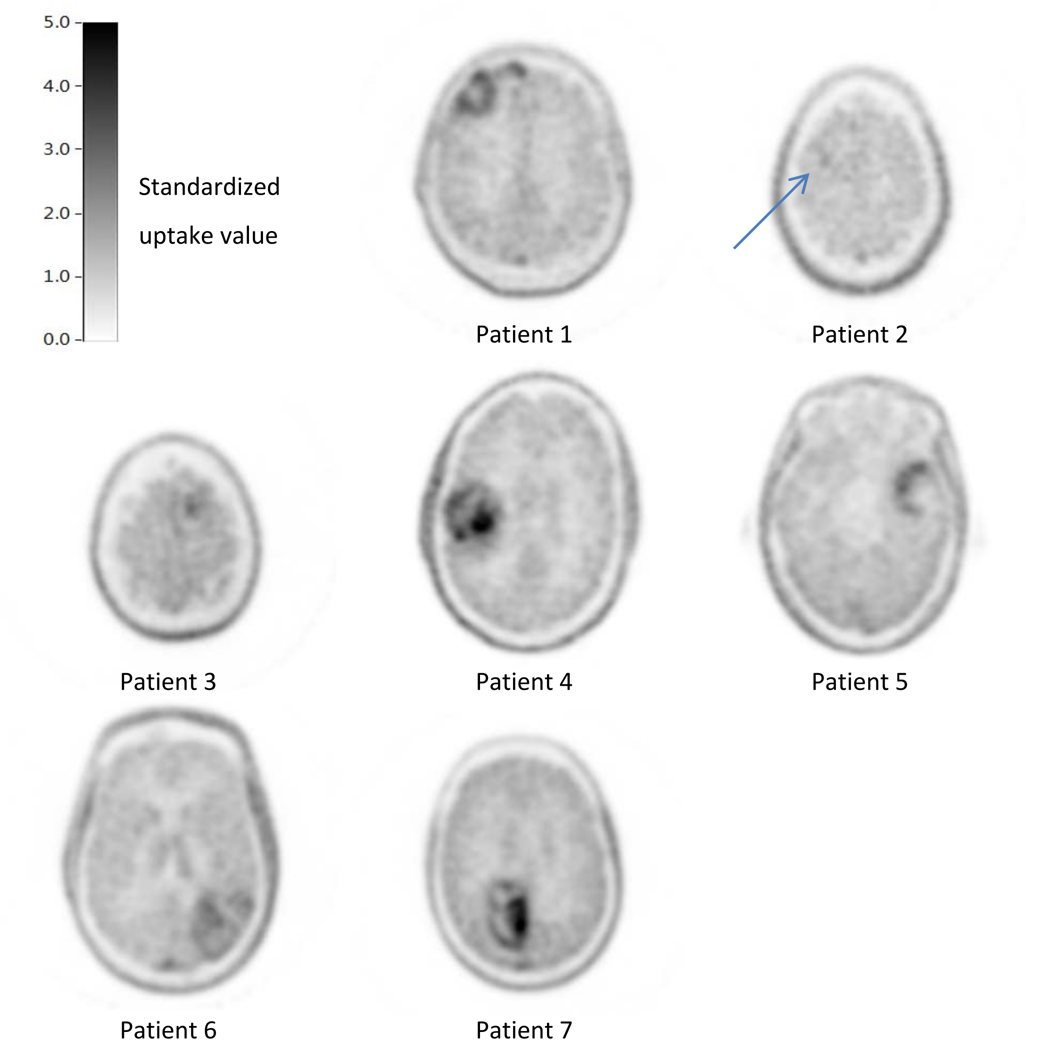

FIGURE S1. Transaxial views of the tumours on 20-40 minutes standardized uptake value maps of [^{18}F]FET.

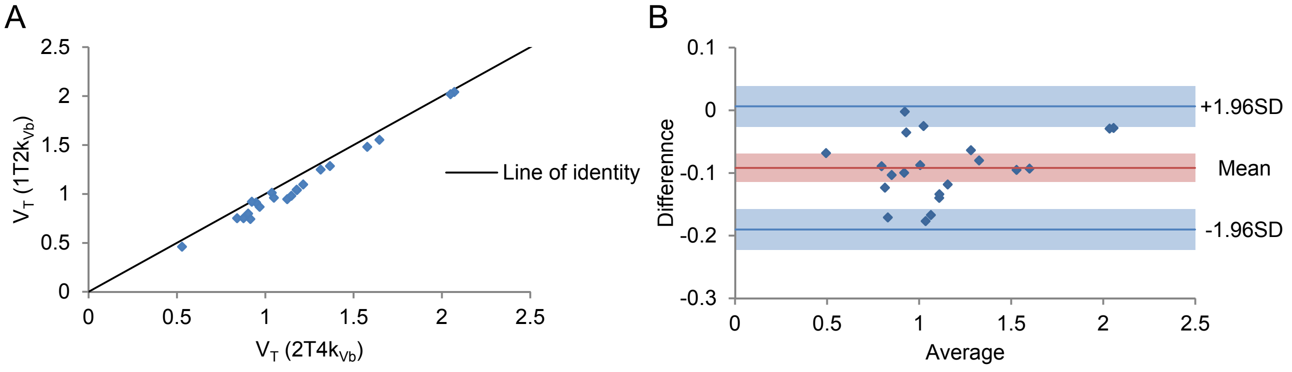

FIGURE S2. Scatter (A) and Bland-Altman plot (B) of volume of distribution, $V_{T,}$ calculated with the $1T2k_{Vb}$ model versus the $2T4k_{Vb}$ model. Shaded areas are 95% confidence intervals.

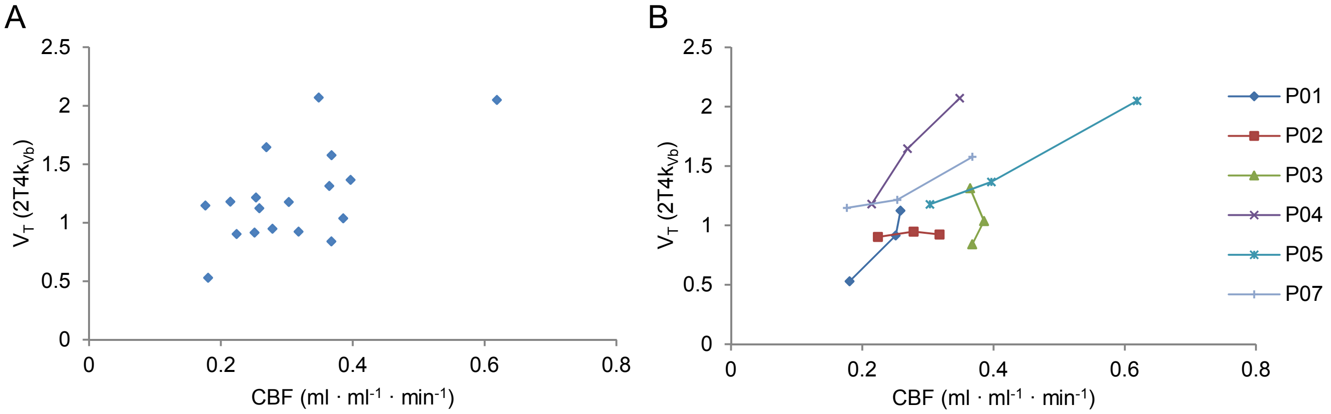

FIGURE S3. Scatterplot of volume of distribution (V_T) versus cerebral blood flow (CBF) (A). The same plot with each patient indicated separately, connecting low, medium, and high VOIs with lines (B).
CBF data was not available for patient 6.

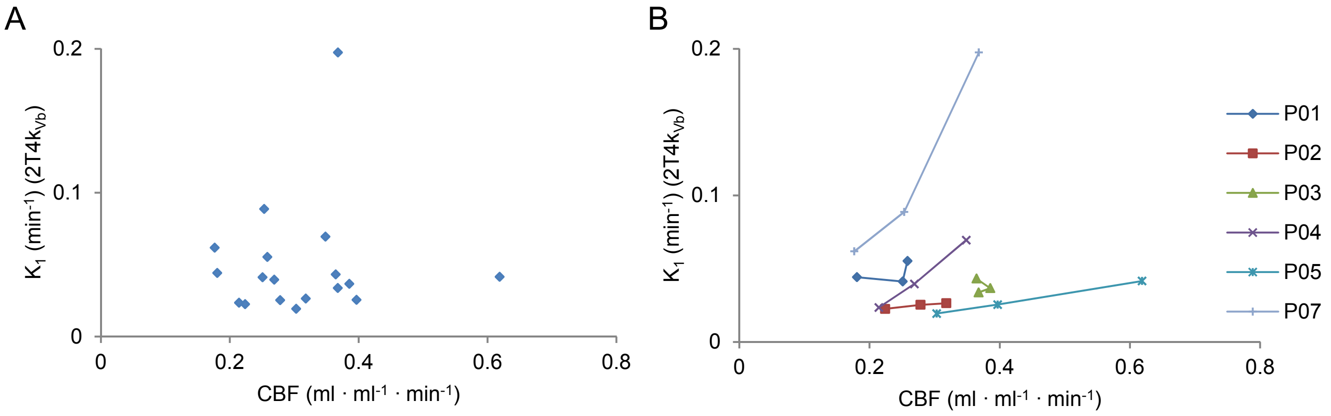

FIGURE S4. Scatterplot of K_1 versus cerebral blood flow (CBF) (A). The same plot with each patient indicated separately, connecting low, medium, and high VOIs with lines (B).
CBF data was not available for patient 6.

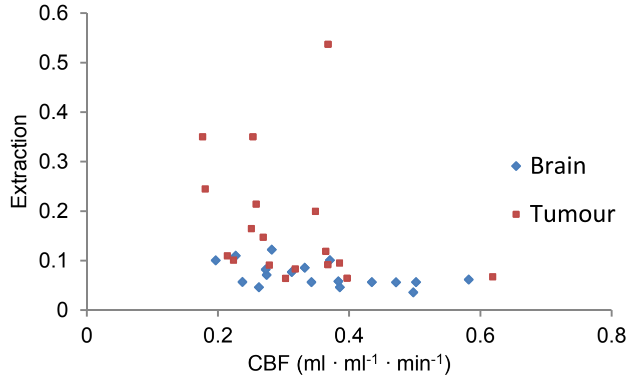

FIGURE S5. Scatterplot of extraction versus cerebral blood flow (CBF).

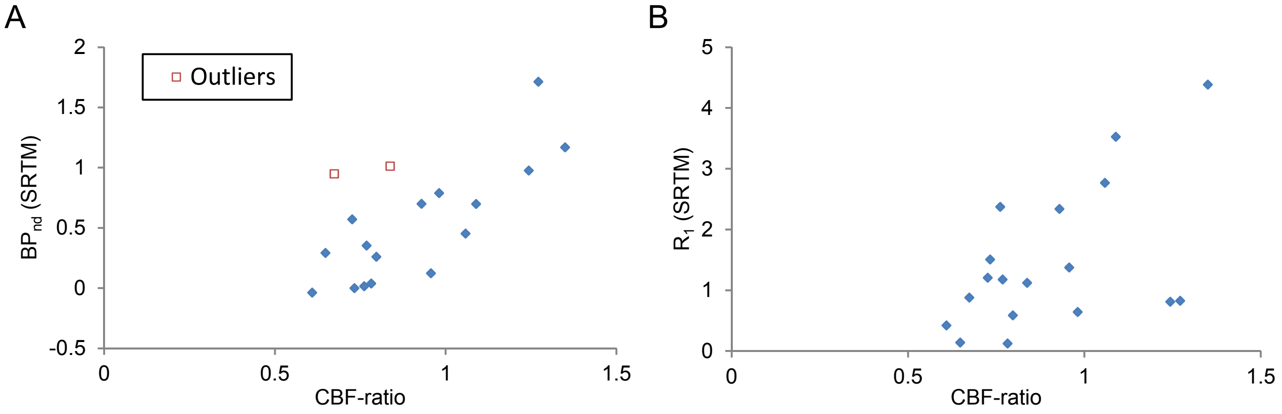

FIGURE S6. Scatterplots of simplified reference tissue model estimates of binding potential (BP_{nd}) (A) and K_1-ratio (R_1) (B) against the cerebral blood flow ratio (CBF-ratio).

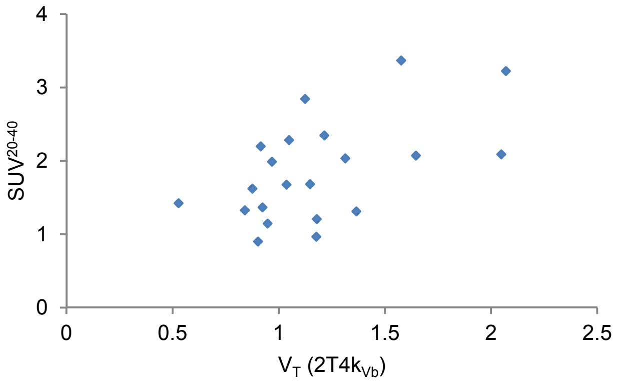

FIGURE S7. Scatter plot of $SUV^{20\text{-}40}$ versus the volume of distribution (V_T) calculated with the $2T4k_{Vb}$ model.
